# Primary hydatid cyst of kidney and ureter with gross hydatiduria: A case report and evaluation of radiological features

**DOI:** 10.4103/0970-1591.38617

**Published:** 2008

**Authors:** Ritesh Mongha, Shrinivas Narayan, Anup K. Kundu

**Affiliations:** Department of Urology, Institute of Postgraduate Medical Education and Research, Kolkata - 700 020, West Bengal, India

**Keywords:** Hydatid, hydatiduria, renal

## Abstract

We report a rare case of echinococcosis, primarily involving the right kidney and ureter, presenting with gross hydatiduria. We also present the salient diagnostic features of renal hydatid.

## INTRODUCTION

Kidney involvement in echinococcosis is extremely rare, constituting only 2-3% of all cases. Primary involvement of the kidney without the involvement of the liver and lungs is even more rare. Hydatiduria accompanies only 10-20% of all cases of renal hydatidosis and is usually microscopic. We present a rare case of primary right renal hydatid having multiple daughter cysts in the upper and mid ureter with gross hydatiduria. The diagnosis of primary hydatid cyst of the kidney, in the absence of hydaturia, is usually radiological as most patients have negative immunological tests. We also present the salient radiological features of primary hydatid cyst of kidney.

## CASE REPORT

A 23-year-old male presented with history of intermittent passage of small, white, balloon-like, grape-sized structures in the urine for the last three months. Abdominal examination did not reveal any palpable lump. Rest of the systemic examination was normal. Gross examination of a single balloon-like structure in the urine revealed a membranous cyst measuring around 2 cm; histopathology showed an outer laminated layer with an inner germinal layer. The laminated structure was consistent with a hydatid cyst.

**Figure 1:**
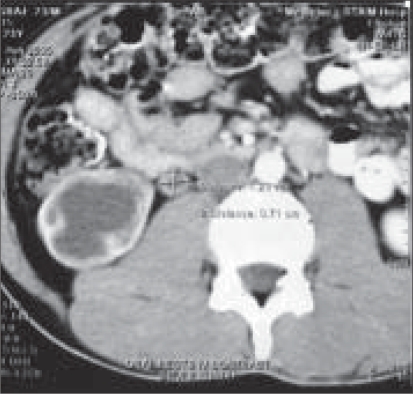
Cystic lesion involving kidney and upper ureter

**Figure 2:**
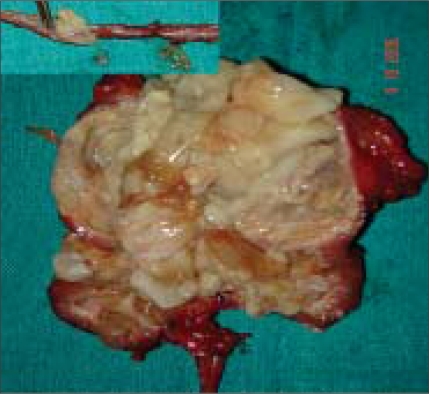
Right kidney completely destroyed by hydatid cysts (inset-multiple daughter cysts in the ureter)

His routine blood investigations were normal with no eosinophilia and normal renal function tests. X-ray chest P-A view was normal. The USG abdomen revealed multiseptate cyst in the right kidney; liver was normal. The CT scan revealed a cystic lesion in the right kidney and upper ureter [Figure 1]. The whole right kidney was replaced by the cystic mass. There was no contrast excretion from the right kidney. Patient was planned for surgery by flank extraperitoneal approach. Per-operatively multiple cysts could be palpated along the length of the ureter. Right nephroureterectomy was done. Patient received four weeks of preoperative albendazole which was continued for eight weeks postoperatively. The resected specimen showed kidney turned into a bag of cysts with multiple daughter cysts in the ureter [Figure 2]. The histopathological examination was consistent with right renal hydatid disease and multiple daughter cysts in the upper and mid-ureter.

## DISCUSSION

Echinococcosis is produced by the larval stage of the Echinococcus tapeworm, *E. granulosus* in this case. Echinococcus belongs to the order Cestoda and the family Taenia. It is about 5 mm long. The adult *E. granulosus* worm resides in the large bowel of foxes and dogs. Man is the intermediate host and gets the disease by ingesting vegetables and water contaminated by the affected dogs. Hydatid disease involves the liver in approximately 75% of cases and the lung in 15%. Secondary involvement due to hematogenous dissemination may be seen in almost any anatomic location. Kidney involvement is extremely rare (2-3%).[1,2] Renal hydatid cysts usually remain asymptomatic for many years. It is not clear how the hydatid embryo reaches the kidney in cases of primary hydatid disease but it is postulated that it must pass through the portal system into the liver and retroperitoneal lymphatics.[3] The hydatid cyst of the kidney is considered closed if all three layers of the cyst i.e. pericyst, ectocyst and endocyst are intact. When the cyst is no longer protected by the third layer i.e. pericyst or by the lining of collecting system it is considered to be an exposed cyst. If all the three layers of the cyst have ruptured resulting in free communication with the calyces and pelvis, it is called an open or communicating cyst. Cystic rupture into the collecting system, causing hydatiduria is pathognomonic, though seen in only 10-20% of renal hydatidosis and is usually microscopic.[4] Gross passage is rather uncommon, but has a tremendous diagnostic utility. Since the cysts passed in the urine are daughter cysts they lack the third layer pericyst, which is contributed by the host around the mother cyst, as was in this case. Eosinophilia is noted in about 50% cases. Serological tests in primary renal hydatidosis are usually negative. Advanced radiological techniques like CT scan and MRI remain the mainstay of diagnosis.

Plain films are usually nonspecific and mostly nonrevealing. A thin rim of calcification delineating a cyst is suggestive of an echinococcal cyst. Ultrasonography helps in the diagnosis of hydatid cysts when the daughter cysts and hydatid sand are demonstrated. On changing the patient's posture under real time, there is shifting of hydatid sand, which may give rise to the "falling snowflake pattern". The accuracy of ultrasound evaluation remains operator-dependent. The CT scan has an accuracy of 98% and sensitivity to demonstrate the daughter cysts. The CT scan usually demonstrates an expansile, hypo-attenuating tumor with a well-defined wall and daughter cysts within the parent cyst. The central cystic part of the lesion has an attenuation of 30-35 HU, in contrast to the much lower attenuation of the fluid in the surrounding cysts (5-15 HU), giving the mass a wheel-like or rosette appearance. Magnetic resonance imaging usually reveals a solitary, high-signal-intensity mass consisting of multiple thin-walled lesions and outlined by a thick, hypointense rim. The high signal intensity is due to the characteristic high fluid content of the mass. The small peripheral cysts are usually hypointense relative to the central component. The MRI shows the cysts adequately, but MRI offers no real advantage over CT scan.[5]

In general, surgery is the treatment of choice in renal hydatid cyst. Kidney-sparing surgery (removal of hydatid cyst with pericystectomy) is possible in most cases (75%). Nephrectomy (25% of cases) must be reserved for destroyed kidney. Very few cases of laparoscopic removal of renal hydatid are reported. There is fear of cyst rupture and dissemination during dissection, entrapment and removal of the hydatid cyst during laparoscopy. Utmost care should be taken during the surgery to prevent spillage and resultant disseminated hydatidosis. Pre and postoperative one-month courses of Albendazole should be considered in order to sterilize the cyst, decrease the chance of anaphylaxis and decrease the tension in the cyst wall (thus reducing the risk of spillage during surgery) and to reduce the recurrence rate postoperatively. During kidney-sparing surgery scolicidal solutions such as hypertonic saline should be used before opening the cavities to kill the daughter cysts and therefore prevent further spread or anaphylactic reaction.
